# 768. Gaining Compliance-Getting Results

**DOI:** 10.1093/ofid/ofab466.965

**Published:** 2021-12-04

**Authors:** Sharon Staton, Janet Dejean

**Affiliations:** Texas Children's Hospital, Peatland, Texas

## Abstract

**Background:**

Compliance with the chlorhexidine gluconate (CHG) daily application component of the CLABSI prevention bundle potentially could negatively affect infection rates. In an attempt to increase CHG compliance, a 3-month trial for soap based CHG bathing was undertaken on two pediatric oncology units with long term central line patients.

**Methods:**

The current bathing process involved 2 steps, a soap and water bath followed one hour later by a CHG wipe. It was time consuming and received complaints from staff and parents resulting in lower documented compliance rates. A one step process was implemented combining skin cleansing and CHG application with one product.

Staff, parents and patients were educated on proper bathing technique. Instruction brochures printed in multiple languages were employed and discussed for education. An electronic survey was developed to collect parent feedback.

**Results:**

The trial was from October - December 2020 and included 25 select patients in the cancer center. Patients and parents provided positive feedback with the new process.

Audits measured both line maintenance and bathing. If one step was missed-than non- compliance with the bundle was noted . Bundle adherence increased with auditors noting that this was due entirely to an increase in bathing compliance. From April to September 2020 prior to implementation of the soap based CHG bathing, CHG compliance on the Stem Cell Transplant Unit (SCTU) averaged 48%. During the three month period after the trial, CHG compliance has averaged 64%. CHG monthly compliance reached 85% by April 2021. In addition, patients compliant with CHG bathing demonstrated a significant reduction in coagulase negative staphylococcus (CoNS ) blood stream infections due to the reduction of CoNS skin colonization. Cost analysis for the one week in the 15 bed BMT unit and 10 HemONC patients showed that the one step soap based CHG was &161.50 and the CHG wipe cost &960,75; a difference of &799.25per week or &41,561.00 annually.

**Conclusion:**

Any infection prevention strategy needs to involve staff and parents for compliance and outcome success.

**Disclosures:**

**All Authors**: No reported disclosures

